# High-Quality Representation Learning Approach to Spatio-Temporal Traffic Speed Data with *L_p,ϵ_*-Norm

**DOI:** 10.3390/e28040435

**Published:** 2026-04-13

**Authors:** Lei Yang, Ziwen Ma, Yikai Hou

**Affiliations:** 1 School of Computer Science and Technology, Chongqing University of Posts and Telecommunications, Chongqing 400065, China; 2 College of Computer and Information Science, Southwest University, Chongqing 400715, China

**Keywords:** *L_p,ϵ_*-norm, latent factorization of tensors, representation learning, spatio-temporal traffic data, intelligent transportation systems

## Abstract

In the realm of intelligent transportation systems (ITS), achieving optimal system performance relies heavily on the acquisition of comprehensive and high-quality spatio-temporal traffic data. In practical data-gathering processes, factors such as sensor malfunctions or communication interruptions often lead to incomplete or missing data records, which in turn substantially hinder the advancement of ITS applications. To address missing spatio-temporal data, a widely adopted paradigm involves the Latent Factorization of Tensors (LFT) model. Traditional LFT frameworks often employ the standard *L*_2_ metric in their learning objective, making them easily affected by abnormal data points. Moreover, impulse noise frequently arises in sensors and communication scenarios. To address these limitations, this paper develops an Adaptive *L_p,ϵ_*-norm-incorporated Latent Factorization of Tensors (*L_p,ϵ_*LFT) model founded on two-fold concepts: (a) constructing a generalized objective function grounded in the *L_p,ϵ_*-norm distance to enhance robustness against outliers; (b) realizing the self-adaptation of model hyper-parameters through a fuzzy controller to enhance model practicality. Experimental evaluations on six traffic speed datasets derived from multiple metropolitan traffic networks demonstrate that the proposed *L_p,ϵ_*LFT model yields significantly higher imputation accuracy and superior computational efficiency compared with seven state-of-the-art approaches.

## 1. Introduction

Big-data-driven intelligent transportation systems (ITSs) frequently involve large-scale and dynamically evolving spatio-temporal networks, such as the real-time traffic interactions among numerous road segments, intersections, and sensing devices within a metropolitan traffic management platform. These intricate interactions can be uniformly represented as a Spatio-temporal Weighted Directed Network (SWDN), where the dynamically weighted links indicate the observed correlations between nodes at each time slot, and each weight quantifies the strength of traffic influence.

**Figure 1:**
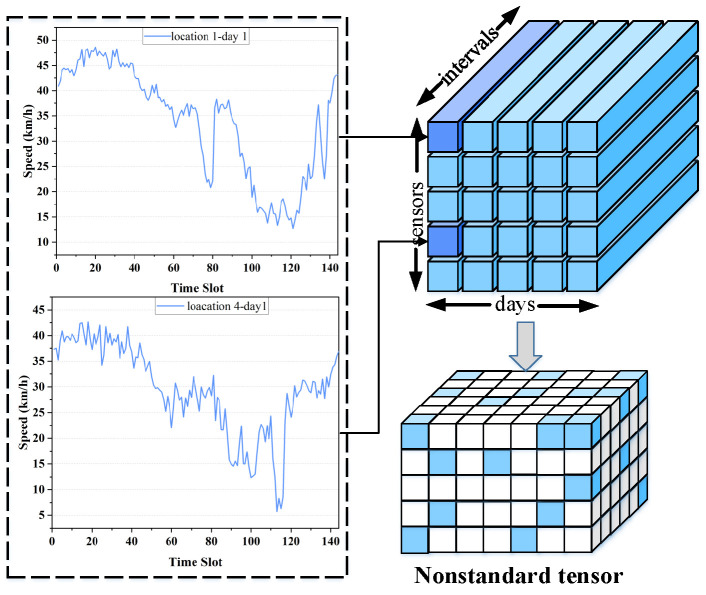
Data distribution of real Guangzhou speed.

Owing to the massive number of sensing terminals and the limitations in data acquisition–such as sensor malfunctions, communication instability, and insufficient deployment–it is practically impossible to obtain complete traffic observations across all spatial and temporal dimensions. Consequently, the structure of an SWDN becomes high-dimensional, incomplete, and unbalanced. A third-order nonstandard tensor [1] is frequently employed to quantify such a network, as it comprehensively characterizes the spatio-temporal correlations of traffic states across multiple locations and times. However, this nonstandard tensor typically contains a large portion of missing entries and is often contaminated by impulse noise arising from sensing and transmission errors [2]. The statistical characteristics of impulse noise are similar to those of outlier behavior, since its long-tailed distribution increases the likelihood of extreme deviations from the mean compared with Gaussian noise. Figure 1 illustrates an example using real-world traffic speed data collected in Guangzhou, China. While most speed observations fall within a normal range, a few values deviate significantly, reflecting inevitable outliers in ITS datasets. Indiscriminate removal of these outliers may lead to the loss of meaningful information, as some extreme values may correspond to real traffic events, such as congestion.

Given the aforementioned challenges, accurately recovering missing spatio-temporal traffic information under impulse noise environments has become a critical yet challenging problem. To address this challenge, researchers have developed LFT models to extract latent representations from nonstandard tensors. By efficiently encoding the partially observed data into a compact latent space, LFT models have achieved promising performance in various spatio-temporal prediction tasks. Yang et al. [3] propose a collaborative Bayesian tensor factorization method for traffic speed prediction that shares a common factor matrix to enable collaboration. Chen et al. [4] introduce a dynamic autoregressive tensor factorization framework in which tensor factorization is seamlessly integrated into the time-varying autoregression for discovering spatial and temporal patterns from the spatio-temporal systems. Zhu et al. [5] introduce a multi-task neural tensor factorization approach to jointly impute traffic speed and volume, utilizing a multi-layer neural network in place of the CP factorization’ inner product, effectively capturing the non-linear relationships among shared attributes. Xing et al. [6] propose a data fusion-based CP tensor factorization model that constructs a four-way tensor through the integration of vehicle data along with cellphone data to impute missing network volume data. Chen et al. [7] proposed a framework for tensor completion that maintains temporal dimensions and enforces non-negativity. The method reconstructs the original tensor using three latent components and applies a Sigmoid activation to guarantee that the recovered traffic data remain non-negative.

Nevertheless, existing LFT methods commonly adopt general *L*_2_-norm-based learning objectives, which assume Gaussian noise distributions and fail to account for impulsive disturbances or outliers prevalent in real-world ITS data [2]. As a result, while they may achieve stable predictions for missing entries, their robustness cannot be guaranteed when impulse noise is present. To mitigate impulsive interference in wireless communication links, many studies have adopted an *L_p_*-norm-based distance metric [8].

Wu et al. [9] incorporating *L*_1_-norm into a prediction’s loss function. Chen et al. [10] combine multiple *L_p_*-norms to obtain the aggregated loss with the hybrid effects by different norms. These two approaches can essentially be regarded as weighted combinations of *L_p_* norms, which inevitably introduce additional hyperparameters to balance the respective contributions. Zeng et al. [8] propose a unified form governed by a single parameter *p* via minimizing the *L_p_*-norm (1≤p<2) of the residual error matrix. To address the non-smoothness and non-convexity of the *L_p_*-norm for (0≤p<1), Li et al. [2] and Zeng et al. [11] employ the alternating direction method of multipliers for *L_p,ϵ_*-norm minimization, but this approach suffers from high computational complexity.

**Figure 2:**
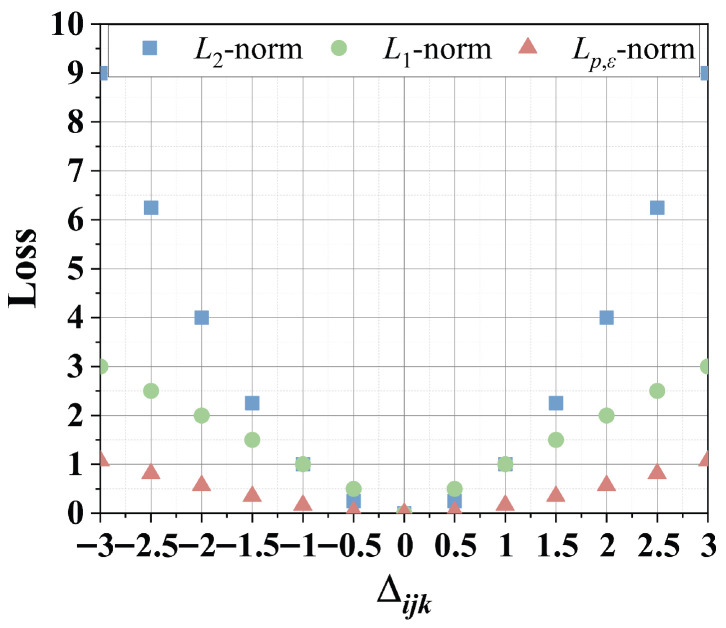
The difference between *L*_2_-norm, *L*_1_-norm, and *L_p,ϵ_*-norm in defining the loss function of an LFT model, where *p* = 0.4 and ϵ = 0.2.

As pointed out in [8,11,12], *L_p,ϵ_*-norm is a more generalized form of distance measurement, with the commonly used *L*_2_-norm representing only a specific case within this broader family. As shown in Figure 2, the distinction between the *L*_2_-norm and *L_p,ϵ_*-norm is illustrated, where $\Delta_{ijk}$ represents the deviation between predicted outputs and the corresponding true labels. It is evident from the figure that, compared to the *L*_2_-norm, the *L_p,ϵ_*-norm exhibits reduced sensitivity to these error terms. This characteristic indicates that the *L_p,ϵ_*-norm is more robust in mitigating the influence of outliers, thereby offering improved tolerance to anomalous data points. Nevertheless, the performance of *L_p,ϵ_*-norm is highly sensitive to the selection of parameters *p* and ϵ, which are inherently data-dependent. These parameters require careful and thorough tuning to achieve satisfactory results. Motivated by these insights, this study formulates a research question aimed at further investigating this direction. The proposed *L_p,ϵ_*LFT models can be distinguished from existing models with respect to the following aspects.

1. It constructs a learning objective grounded in the *L_p,ϵ_*-norm. As a result, it achieves accurate reconstruction of missing values from partially observed traffic data.
2. The model’s parameters are automatically adjusted using a fuzzy controller for high scalability.

We outline the structure of this paper as follows: Section 2 surveys the related literature, Section 3 introduces the foundational conceptions, Section 4 details the proposed methodology, Section 5 examines the experimental results thoroughly, and Section 6 presents the summary of the study.

## 2. Related Work

Over the past few years, various methods have been developed to accurately recover traffic data from multiple perspectives. These methods can be broadly classified into three main types.

A model based on matrix factorization. These models transform a data imputation problem into a matrix factorization task. Specifically, they decompose the target matrix into low-rank matrices using a particular decomposition technique. Yang et al. [13] introduce a latent factor analysis model incorporating a temporal regularization constraint that utilizes first-order temporal differences to capture time-series dynamics. Xu et al. [14] propose a spatio-temporal low-rank technique with Hessian regularization to impute traffic data, where temporal and spatial regularization terms are built using a Toeplitz matrix and a Hessian matrix. Sure et al. [15] develop the augmented Lagrangian sparsity-aware matrix factorization and constraint-enhanced matrix completion methods for traffic matrix reconstruction, which incorporate a graph adjacency matrix and a Toeplitz matrix as spatial and temporal regularization.

A tensor decomposition-based method. These models transform the original time series matrix into a third-order tensor by incorporating an additional “time slot” dimension. Chen et al. [16] propose a fourth-order dimension preserved tensor completion model that incorporates both temporal and non-negative constraints to capture spatio-temporal information. Chen et al. [17] propose a nonconvex smoothly clipped absolute deviation penalty tailored for tensors to approximate tensor rank and integrate the deep plug-and-play prior into the low-rank tensor completion (LRTC) model. Baggag et al. [18] present a temporally regularized tensor factorization framework for estimating missing values, which incorporates not only a graph Laplacian spatial regularization term but also an autoregressive temporal regularization term for tensor decompositions. Shu et al. [19] propose a 3D spatio-temporal transform LRTC method, which uses the truncated tensor nuclear norm on the spatio-temporal feature tensors.

A neural network-based method. Wei et al. [20] introduce an imputation approach based on graph convolution with self-attention, which combines diffusion graph convolution and an attention mechanism to model spatio-temporal patterns in traffic data. Zhang et al. [21] present a traffic data completion framework using graph transformers, where graph neural networks capture spatial correlations while Transformer modules analyze temporal sequences. Zhang et al. [22] design an attention-driven spatio-temporal network model for urban flow imputation. Zhang et al. [23] propose a residual network that fuses graph convolution with multi-head self-attention to encode both local structures and contextual information for traffic data recovery. Xu et al. [24] propose a multi-level graph convolutional neural network that captures spatio-temporal patterns in traffic data, leveraging temporal correlation clustering along with an attention mechanism to extract relevant spatio-temporal information.

However, despite variations in model architectures and learning strategies, the aforementioned imputation methods commonly employ the *L*_2_-norm loss function to reduce the difference between predicted values and the ground truth. Notably, this norm is known to be affected by extreme values [25,26]. Therefore, they are prone to substantial performance loss when the data deviate from their underlying distributional assumptions. In this study, the *L_p,ϵ_*LFT model incorporates a *L_p,ϵ_*-norm to establish a robust learning objective and utilizes a fuzzy controller for parameter adaption.

## 3. Preliminaries

### 3.1. Data Recovery Problem in ITS

Table 1 lists the symbols used throughout this study. Data collected from ITS are often incomplete due to factors such as sensor malfunctions, communication failures, limited coverage, and human errors. One of the main challenges in recovering ITS data is to estimate the missing values from the observations that are available. In the next section, we formally define this problem.

**Definition 1**  (*Data Recovery Problem in ITS*)**.** *Let I, J, and K represent three extensive sets representing sensors, time intervals, and days, respectively. The measured traffic speeds are arranged as a third-order tensor $Y^{\left|I|\times |J|\times |K\right|}$, with each entry $y_{ijk}$ corresponding to the speed recorded by sensor i∈I during time interval j∈J on day k∈K. Let* Λ *indicate the set of known elements in Y, and*  Γ *represent the missing entries.*

### 3.2. *Latent Factorization of Tensors (LFT)*

Consider a third-order tensor $Y^{\left|I|\times |J|\times |K\right|}$. The LFT model aims to estimate Y by approximating it as a rank-R tensor ${\hat{Y}}^{\left|I|\times |J|\times |K\right|}\in R$.

**Definition** **2** (*LFT Model*)**.** *Given a tensor Y, the LFT model approximates it as a sum of R rank-one tensors: $\hat{Y}=\sum_{r=1}^{R}X_{r}$, where each $X_{r}^{\left|I|\times |J|\times |K\right|}$ is a rank-one tensor. Before proceeding, we clarify the definition of a rank-one tensor.*

**Definition** **3** (*Rank-one Tensor*)**.** *A rank-one tensor $X_{r}$ can be represented as the outer product of three latent factor (LF) vectors [27,28]: $X_{r}=u_{r}\circ s_{r}\circ t_{r}$, where $u_{r}^{\left|I\right|}$, $s_{r}^{\left|J\right|}$, and $t_{r}^{\left|K\right|}$ are LF vectors corresponding to sensors, time intervals, and days, respectively.*

These LF vectors constitute three LF matrices $U^{|I|\times R}$, $S^{|J|\times R}$, and $T^{|K|\times R}$. Each entry of $X_{r}$, denoted $x_{ijk}^{\left(r\right)}$, can then be calculated as:

(1) $$x_{ijk}^{\left(r\right)}=u_{ir}s_{jr}t_{kr}.$$

Therefore, the value ${\hat{y}}_{ijk}$ obtained in the reconstructed tensor $\hat{Y}$ can be expressed in the following manner:

(2) $${\hat{y}}_{ijk}=\sum_{r=1}^{R}x_{ijk}^{\left(r\right)}=\sum_{r=1}^{R}u_{ir}s_{jr}t_{kr}.$$

To obtain the LF matrices U, S, and T, one typically measures the reconstruction error between the original tensor and its low-rank approximation $\hat{Y}$ using the *L*_2_-norm [29,30]. Given that the traffic tensor Y is sparsely observed [31], the LFT loss function is defined only over the observed subset Λ, which is consistent with density-oriented modeling principles [32]. There, the optimization target can be expressed in terms of the Euclidean distance as:

(3) $$\begin{array}{c} \varepsilon =\frac{1}{2}{\sum_{y_{ijk}\in \Lambda }\left(y_{ijk}-{\hat{y}}_{ijk}\right)}^{2}=\frac{1}{2}{\sum_{y_{ijk}\in \Lambda }\left(y_{ijk}-\sum_{r=1}^{R}u_{ir}s_{jr}t_{kr}\right)}^{2}. \end{array}$$

The irregular distribution of these entries in Y, together with the model’s sensitivity to the initial settings of U, S, and T, renders problem (3) ill-posed. Introducing Tikhonov regularization improves both model robustness and generalization, yielding

(4) $$\begin{array}{c} \varepsilon =\frac{1}{2}\sum_{y_{ijk}\in \Lambda }{\left(y_{ijk}-\sum_{r=1}^{R}u_{ir}s_{jr}t_{kr}\right)}^{2}+\frac{\lambda }{2}\sum_{y_{ijk}\in \Lambda }\left(\sum_{r=1}^{R}\left(u_{ir}^{2}+s_{jr}^{2}+t_{kr}^{2}\right)\right). \end{array}$$

The learning process for the desired LF $u_{ir}$ is formulated using SGD as follows:

(5) $$\begin{array}{c} \underset{U}{\arg\min\varepsilon }\;\underset{\Rightarrow }{SGD}\forall y_{ijk}\in \Lambda :u_{ir}^{n+1}\leftarrow u_{ir}^{n}-\eta \cdot \frac{\partial \varepsilon_{ijk}}{\partial u_{ir}^{n}}=u_{ir}^{n}-\eta \left(-e_{ijk}s_{jr}^{n}t_{kr}^{n}+\lambda u_{ir}^{n}\right), \end{array}$$

where $e_{ijk}=y_{ijk}-{\hat{y}}_{ijk}$ represents the instant error in $y_{ijk}$, and the updates for $s_{jr}$ and $t_{kr}$ follow analogously. Using Equation (5) allows the SGD-based LFT model to effectively uncover latent patterns in the tensor.

### 3.3. Fuzzy Control

Fuzzy control is a well-established control strategy recognized for its ability to effectively manage uncertainty and imprecision. In traditional control methods, system inputs and outputs are typically represented using precise numerical values. In contrast, fuzzy control incorporates the principles of fuzzy logic, enabling systems to effectively address uncertainties inherent in real-world environments. By integrating fuzzy logic, fuzzy control allows systems to adapt more efficiently to the inherent complexity and unpredictability of real-world processes.

## 4. Proposed *L_p,ϵ_*LFT Model

### 4.1. Generalized Objective Function

To improve resistance to outliers, we modify Equation (4) by substituting the *L*_2_-norm with the *L_p_*-norm, as follows:

(6) $$\begin{array}{c} \varepsilon ={∥E∥}_{p}={\left(\sum_{y_{ijk}\in \Lambda }{\left|y_{ijk}-\sum_{r=1}^{R}u_{ir}s_{jr}t_{kr}\right|}^{p}\right)}^{\frac{1}{p}}+\frac{\lambda }{2}\sum_{y_{ijk}\in \Lambda }\left(\sum_{r=1}^{R}\left(u_{ir}^{2}+s_{jr}^{2}+t_{kr}^{2}\right)\right), \end{array}$$

where ${\left||.|\right|}_{p}$ denotes the element-wise *L_p_*-norm of a tensor, where E represents the error tensor. In the special case where *p* = 1, (6) corresponds to the *L*_1_-norm, which minimizes the sum of the absolute errors.

However, the nonsmoothness and nonconvexity of the *L_p_*-norm, particularly for 0<p≤2, introduce significant challenges in the optimization process. Hence, it is essential to incorporate ϵ>0 into it for adjusting the convexity of *L_p,ϵ_*-norm, yielding

(7) $$\begin{array}{c} \varepsilon ={∥E∥}_{p,\varepsilon }={\left(\sum_{y_{ijk}\in \Lambda }{\left({\Delta_{ijk}}^{2}+\varepsilon^{2}\right)}^{p/2}-\varepsilon^{p}\right)}^{\frac{1}{p}}+\frac{\lambda }{2}\sum_{y_{ijk}\in \Lambda }\left(\sum_{r=1}^{R}\left(u_{ir}^{2}+s_{jr}^{2}+t_{kr}^{2}\right)\right), \end{array}$$

where $\Delta_{ijk}=y_{ijk}-\sum_{r=1}^{R}u_{ir}s_{jr}t_{kr}$.

We analyze the relationship between the *L_p,ϵ_*-norm and the standard *L_p_*-norm. It can be shown that the *L_p,ϵ_*-norm serves as a lower bound for the *L_p_*-norm for 0<p≤2. This property implies that the proposed norm provides a smoother approximation of the *L_p_*-norm, especially around zero. Such smoothing helps alleviate numerical instability caused by the non-differentiability or sharp curvature of the *L_p_*-norm, which is beneficial for optimization. In particular, the introduction of the parameter ϵ avoids singularities and leads to more stable gradient behavior. As a result, the optimization process becomes more robust and less sensitive to noise or initialization. For completeness, a proof of this property is provided in Appendix A.

### 4.2. SGD-Based Learning Rules

An SGD algorithm is employed to solve (7) with respect to U, S, and T, as it is highly effective in optimizing the desired LFs

(8) p≠1:uirn+1←uirn−ηΔijk2+ε2pp22−εp11pp−1·Δijk·Δijk2+ε2pp2−12−1−sjrntkrn+λuirnsjrn+1←sjrn−ηΔijk2+ε2pp22−εp11pp−1·Δijk·Δijk2+ε2pp2−12−1−uirntkrn+λsjrntkrn+1←tkrn−ηΔijk2+ε2pp22−εp11pp−1·Δijk·Δijk2+ε2pp2−12−1−uirnsjrn+λtkrnp=1:uirn+1←uirn−ηsignΔijk·−sjrntkrn+λuirnsjrn+1←sjrn−ηsignΔijk·−uirntkrn+λsjrntkrn+1←tkrn−ηsignΔijk·−uirnsjrn+λtkrn

where *n* and (*n* + 1) denote the *n*th and the (*n* + 1)-th update points of the LF matrices, sign(.) denotes the sign function, η represents the learning rate, λ denotes the regularization coefficient, respectively. Notably, for *p* = 1, Equation (7) degenerates into the *L*_1_-norm. However, as the *L*_1_-norm is non-differentiable at zero, we extend it into the differentiable form given in Equation (8) for the case of *p* = 1. With (8), the learning scheme arrived.

### 4.3. Fuzzy Reasoning Rule Designing

Since (8) relies on four hyper-parameters, namely *p*, ϵ, η, and λ, it is crucial to make them self-adaptive; otherwise, their tuning would be computationally expensive. According to previous studies, an adaptive system employing fuzzy control can not only enhance the reliability of the control process but also achieve superior performance. Therefore, a fuzzy control-based hyper-parameters adaptation mechanism is proposed for *L_p,ϵ_*LFT. To this end, a one-dimensional fuzzy control approach is employed. During the *t*-th training iteration, the input variable of the fuzzy reasoning rule is formulated as follows.

(9) Dt=ω∑yijk∈Ψyijk−y^ijk2/∑yijk∈Λyijk−y^ijk2ΨΨ+1−ω∑yijk∈Ψyijk−y^ijkabs∑yijk∈Ψyijk−y^ijkabsΨ/Ψ

(10) $$I^{\left(t\right)}=D^{\left(t-1\right)}-D^{\left(t\right)}$$

**Fuzzing reasoning.** Based on the inherent characteristics of *L_p,ϵ_*LFT and SGD methods, the fuzzy inference rules for the parameters *p*, ϵ, η, and λ are formulated as follows:

1. As training progresses, the model error gradually decreases and the parameters progressively approach their optimal value. At this stage, it becomes crucial to reduce the step size η to mitigate oscillations around the optimal solution, thereby enhancing the stability of the optimization process. To prevent premature convergence to a local optimum and the consequent model instability, it is advisable to progressively decrease the step size η as the model error diminishes throughout the training process.
2. As model training progresses and the error diminishes, gradually increasing the regularization coefficient λ is effective in alleviating overfitting and enhancing the model’s generalization capacity. During the initial training phases, when the error remains relatively high, a smaller λ allows more flexible model parameter adjustments, thereby accelerating error reduction. Conversely, in the later stages of training, as the error declines, increasing λ effectively regulates model complexity, thereby preventing overfitting to noise within the training data and enhancing performance and robustness on unseen datasets.
3. In the early stages of training, when the model error remains relatively high and the influence of outliers on the overall trend is limited, maintaining a relatively large *p* enables the model to adapt with greater flexibility. This facilitates rapid identification of the underlying data patterns without excessively suppressing informative signals. As training progresses and the model error decreases, the relative impact of outliers becomes more pronounced. If *p* remains large, the model may be negatively affected by outliers, leading to fluctuations and instability in the vicinity of the optimal solution. Therefore, in the later stages of training, a gradual reduction of *p* plays a crucial role in suppressing the influence of outliers, thereby enhancing the robustness and stability of the model and mitigating the risk of noise driving the parameters away from the optimal solution.
4. In the early stages of training, when the model error remains relatively high and achieving rapid yet stable progress is a priority, maintaining a relatively large ϵ is beneficial. A large ϵ enhances the smoothness and convexity of the *L_p,ϵ_*-norm, leading to stable gradient behavior and facilitating efficient optimization without being trapped by sharp or ill-conditioned penalty geometries. As training progresses and the model error decreases, fidelity to the true *p*-norm becomes increasingly important for suppressing the influence of outliers. Therefore, in the latter stages of training, it is advisable to gradually reduce ϵ, thereby ensuring that the penalty more closely approximates the original *p*-norm and enforces stronger robustness. This strategy balances the final model’s expressivity and robustness against numerical stability by starting with larger smoothing to accelerate and stabilize convergence and ending with smaller smoothing to recover the desired *p*-norm behavior.

Table 2 illustrates a designed fuzzy rule, in which a triangular membership function is employed to update the hyper-parameters as follows:

ifIt<I1:deg1=0,deg2=1pt=deg2×p1,εt=deg2×ε1ηt=deg2×η1,λt=deg2×λ1.ifI1≤It<I2:deg1=It−I1It−I1I2−I1I2−I1deg2=I2−ItI2−ItI2−I1I2−I1pt=deg1×p2+deg2×p1εt=deg1×ε2+deg2×ε1ηt=deg1×η2+deg2×η1λt=deg1×λ2+deg2×λ1.……

ifI4≤It<I5:deg1=It−I4It−I4I5−I4I5−I4deg2=I5−ItI5−ItI5−I4I5−I4pt=deg1× p5+deg2× p4εt=deg1× ε5+deg2× ε4ηt=deg1× η5+deg2× η4λt=deg1× λ5+deg2× λ4.

(11) $$\begin{array}{c} if\left(I^{\left(t\right)}\geq I_{5}\right):\left\{\begin{array}{c} \deg_{1}=0,\deg_{2}=1\\ p^{t}=\deg_{2}\times \;p_{5}\\ \varepsilon^{t}=\deg_{2}\times \;\varepsilon_{5}\\ \eta^{t}=\deg_{2}\times \;\eta_{5}\\ \lambda^{t}=\deg_{2}\times \;\lambda_{5} \end{array}\right.. \end{array}$$

where $p^{t}$$,\epsilon^{t}$, $\eta^{t}$, and $\lambda^{t}$ represent the state values of *p*. ϵ, η, and λ at the *t*-th updates, respectively. The parameter update strategy in (11) is designed as an adaptive mechanism driven by the reduction of reconstruction error, as quantified by $I^{\left(t\right)}$. Specifically, $I^{\left(t\right)}$ measures the improvement between two consecutive iterations, reflecting the current convergence behavior of the model. When the improvement is large, it indicates that the model is still in a rapidly evolving stage. In this case, a more conservative parameter setting is adopted to maintain stability and avoid undesirable oscillations. As the improvement becomes smaller, the optimization gradually approaches convergence, and the parameters are adjusted to enhance sparsity and regularization strength for more refined reconstruction. Since the parameter configurations are defined at discrete levels, (11) employs a piecewise linear interpolation scheme to ensure smooth transitions between adjacent settings. This avoids abrupt parameter changes and contributes to stable convergence. Overall, this strategy enables the model to adapt its parameter configuration according to the convergence progress, improving both robustness and reconstruction performance. By combining (11) and (8), the learning scheme of the *L_p,ϵ_*LFT is derived, incorporating a fuzzy-based hyper-parameter adaptation mechanism.

### 4.4. Algorithm Design and Analysis

Building on the aforementioned observations, we propose Algorithm 1 for the *L_p,ϵ_*LFT model, the detailed procedure of which is presented in Algorithm 1. More specifically, the proposed algorithm consists of the following five steps:
**Algorithm 1** *L_p,ϵ_*LFT**Operation**                    **Cost**  1:Input: Λ,R  2:Initialize U, S, T             Θ((|I|+|J|+|K|)×R)  3:Initialize *p*, ϵ, η,λ,n=1, N=1000          Θ(1)  4:**while**n≤N**and** not converge **do**           ×n  5:     **for** each $\forall y_{i,j,k}\in \Lambda$**do**               ×|Λ|  6:           Update (U, S, T) based on (8)         Θ(3R)  7:     **end for**  8:     Computing $D^{\left(t\right)}$ and $I^{\left(t\right)}$ based on (9) and (10)   Θ(Ψ)  9:     Computing deg1 and deg2 based on (11)10:     Computing $\eta^{\left(t\right)},\lambda^{\left(t\right)}$ based on (11) and Table 2    Θ(1)11:     n=n+1                     Θ(1)12:**end while**13:**Output:** U, S, T

*Step 1:* The LF matrices are initialized with uniformly distributed non-negative random values in the interval [0,2.0], following previous studies. Note that the bias introduced by random initialization can be mitigated by training the model multiple times. The hyper-parameters are set empirically based on prior experience.

*Step 2:* The LF matrices are updated according to (8) using the initial hyper-parameters.

*Step 3:* Following the fuzzy control rule defined in (11), the hyper-parameters are updated based on the discrepancy observed in the first training iteration.

*Step 4:* The LF matrices are subsequently updated according to (8) using the updated hyper-parameters obtained in Step 3.

*Step 5:* Determine whether the *L_p,ϵ_*LFT model satisfies the convergence criteria; that is, either the iteration count *n* reaches the predefined maximum *N*, or the RMSE/MAE difference between two consecutive iterations falls below a predefined threshold. If the convergence criteria are satisfied, the final LF matrices are output; Otherwise, the process returns to Step 3.

It is important to note that Algorithm 1 provides a summary of the time complexity for each individual step. Consequently, the overall computational complexity of Algorithm 1 can be determined:

(12) $$\begin{array}{c} C=\Theta ((|I|+|J|+|K|)\times R)+\Theta (n\times |\Lambda |\times R)+\Theta (n\times |\Psi |)+\Theta (1)\approx \Theta (n\times |\Lambda |\times R) \end{array}$$

Its storage complexity is primarily influenced by three factors: (a) the *L_p,ϵ_*LFT model’s parameter set {U, S, T, }; (b) entries in Y and $\hat{Y}$ corresponding to Λ; and (c) caching the auxiliary vector to maintain the past errors costing Θ(Λ). Thus, the storage complexity of Algorithm 1 is expressed as:

(13) $$\begin{array}{c} S=\Theta ((|I|+|J|+|K|)\times R+2|\Lambda |)\approx \Theta ((|I|+|J|+|K|)\times R+|\Lambda |) \end{array}$$

Therefore, the storage complexity of the *L_p,ϵ_*LFT model can be considered linear with respect to the known entry count of the tensor and its LF matrices.

## 5. Experimental Results and Analysis

### 5.1. General Settings

**Datasets**: An empirical study was conducted using publicly available traffic speed datasets from six major urban areas, namely Guangzhou (China), Seattle, New York, Los Angeles, San Francisco (USA), and Berlin (Germany). The corresponding descriptions of these datasets are presented below.
**D1: Guangzhou Traffic Speed Dataset** [33]. These dataset contains measurements collected from 214 monitoring devices in Guangzhou, China, recorded at 10 min intervals over a 61-day period (1 August–30 September 2016).
**D2: Seattle Dataset**, obtained from the Uber movement project (https://github.com/xinychen/tracebase?tab=readme-ov-file, accessed on 7 January 2026), contains traffic speed data collected from 20,833 detectors in Seattle, WA, USA, recorded hourly over the period 1–31 January 2020.
**D3: New York Speed Dataset**, (https://www.kaggle.com/datasets/crailtap/nyc-real-time-traffic-speed-data-feed, accessed on 7 January 2026 ) includes data collected from 135 monitoring devices in New York, USA, over a 73 days period (1 October–12 December 2022), sampled every five minutes.
**D4: METR-LA Speed Datasets** [34] comprises traffic speed data from 207 detectors in Los Angeles County, USA, recorded every five minutes over the period 1 March–30 June 2012.
**D5: PeMS-BAY Speed Datasets** [34] contains traffic speed information from 325 detectors in the San Francisco Bay Area, California, recorded every five minutes over the period 1 January–30 June 2017.
**D6: Berlin Traffic Speed Dataset**, obtained from the Uber Movement Project (https://github.com/xinychen/tracebase?tab=readme-ov-file, accessed on 7 January 2026), contains traffic speed information from 12,416 detectors in Berlin, Germany, recorded hourly over the period 1–31 January 2020.

Table 3 provides a concise overview of six datasets, enabling a visual comparison.

To minimize potential bias, each dataset is split at random into three non-overlapping parts: a training set K, a validation set Ψ, and a test set Ω, with a proportion of 7:1:2. The model undergoes training on K, validation on Ψ, and final performance measurement on Ω. To mitigate randomness from data splitting, this entire procedure is carried out 20 times, generating multiple sets of experimental result. The mean and standard deviation are then computed to evaluate result stability.

For every model, training will stop if either (1) 1000 iterations have been completed, or (2) the reduction in validation error between two consecutive iterations is less than $10^{-5}$.

**Model Settings**: To achieve dependable outcomes, the experimental setup follows these strategies:

1. In each experiment on the same dataset, the LF matrices are set with identical initial values, which helps reduce the influence of initialization bias.
2. Based on the empirical values obtained from the majority of related studies, we defines the fuzzy rules table in detail as Table 4.
3. The LF space dimension R is consistently fixed at 20 for all models. This choice balances computational cost and the ability to learn effective representation, following the configuration reported in [35].
4. To evaluate the sensitivity of the *L_p,ϵ_*LFT model, we conduct a grid search over the hyper-parameter space: p∈[0.2,2], ϵ∈[0.1,1], $\eta \in [2^{-11},2^{-7}]$, $\lambda \in [2^{-10},2^{-6}]$.

**Evaluation Metric**: We evaluate the model’s performance in tensor representation learning by calculating reconstruction accuracy using two metrics: Root Mean Squared Error (RMSE) and Mean Absolute Error (MAE):

(14) RMSE=∑yijk∈Ωyijk−y^ijk2∑yijk∈Ωyijk−y^ijk2ΩΩMAE=∑yijk∈Ωyijk−y^ijk∑yijk∈Ωyijk−y^ijkΩΩ.

Smaller values of RMSE and MAE correspond to more precise predictions of missing tensor entries, demonstrating enhanced representation learning capability.

### 5.2. Parameter Sensitivity Test

**Figure 3:**
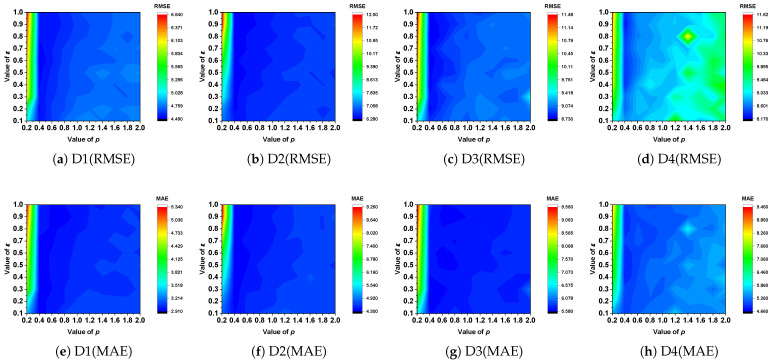
Performance of *L_p,ϵ_*LFT_manual_ varies as *p* and ϵ vary on D1–4.

**Figure 4:**
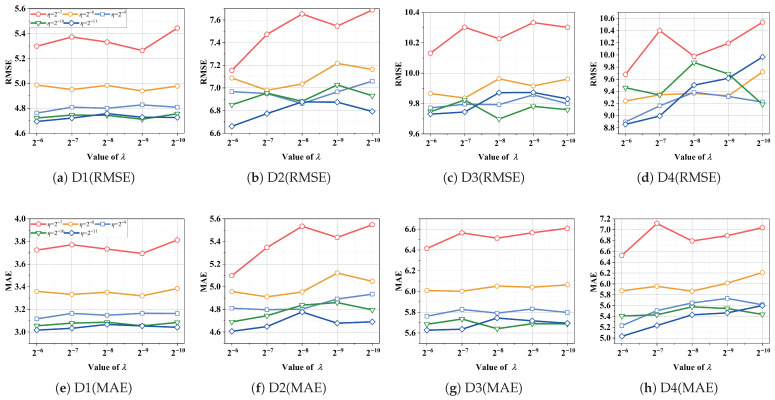
Performance of *L_p,ϵ_*LFT_manual_ varies as η and λ vary on D1–4.

(1) *Effects of p and* ϵ: To evaluate the sensitivity of *L_p,ϵ_*LFT’s performance, we eliminate its parameter adaptation mechanism. We refer to the manually tuned *L_p,ϵ_*LFT as *L_p,ϵ_*LFT_manual_. Initially, we assess its estimation error under varying *p* and ϵ. According to Section 4.1, *L_p,ϵ_*-norm reduces to a standard *L*_1_-norm when *p* = 1, ϵ = 0. Figure 3 illustrates the RMSE and MAE of *L_p,ϵ_*LFT_manual_ under varying *p* and ϵ. Table 5 and Table 6 report the lowest RMSE and MAE obtained at the optimal selections of *p* and ϵ, compared with their corresponding values under the *L*_1_-norm and *L*_2_-norm. In order to verify the statistical significance of the performance improvement of the *L_p,ϵ_*-norm, we performed the Friedman test, win/loss count, and signed rank test. Based on these experimental results, key conclusions are drawn.

1. Be carefully selecting appropriate values for *p* and ϵ, *L_p,ϵ_*LFT_manual_ is able to learn the nonstandard tensor with higher accuracy compared to the commonly adopted *L*_1_-norm and *L*_2_-norm. As shown in Table 5 and Figure 3c, on D3, *L_p,ϵ_*LFT_manual_ achieves the lowest RMSE of 8.7366 when *p* = 0.4 and ϵ = 0.8. In contrast, the standard *L*_2_-norm, achieves an RMSE of 9.1774. Compared with the carefully tuned *L_p,ϵ_*-norm, the performance improvement is 4.80% in terms of RMSE. Analogous phenomena are observed in MAE and across datasets D1-D6. The results show that the *L_p,ϵ_*-norm achieved the lowest value in the F-rank value, confirming the significant advantage of the *L_p,ϵ_*-norm compared to *L*_1_/*L*_2_-norm, and the results of the signed rank test show that the performance difference between the *L_p,ϵ_*-norm and the *L*_1_/*L*_2_-norm is statistically significant (*p*-value < 0.05). These results demonstrate that the *L_p,ϵ_*-norm provides stronger representative capacity compared to the *L*_2_-norm when constructing the learning objective of an LFT model.
2. The optimal ϵ depends on the specific dataset. As illustrated in Table 5, when ϵ is set to 0.6 on D2, *L_p,ϵ_*LFT_manual_ achieves its lowest RMSE of 6.2802. Meanwhile, the optimal RMSE of 8.7366 is achieved when ϵ is adjusted to 0.8 on D3. Moreover, when ϵ becomes too small, the nonsmoothness and nonconvexity of the *L_p,ϵ_*-norm pose challenges to optimization.

(2) *Effects of* η *and* λ: Section 4 demonstrates that the performance of *L_p,ϵ_*LFT_manual_ is also influenced by η and λ. To specifically investigate their impacts, we fix *p* = 1 and ϵ = 0 to validate the effects of η and λ. Figure 4 illustrates the variations in RMSE and MAE corresponding to different settings of η and λ. It is evident from Figure 4 that selecting optimal η and λ is critical for achieving optimal performance of *L_p,ϵ_*LFT_manual_. For instance, as shown in Figure 4a, *L_p,ϵ_*LFT_manual_ achieves the lowest RMSE of 4.6946 with $\eta =2^{-11}$ and $\lambda =2^{-6}$ on D1. Conversely, it records the highest RMSE of 5.4437 with $\eta =2^{-7}$ and $\lambda =2^{-10}$. The performance difference is 13.76%.

### 5.3. Outlier Data Sensitivity Test

In this section, we evaluate the performance of an *L_p,ϵ_*LFT model in comparison with a standard LFT model under datasets containing outliers. Specifically, LFT (*L*2) is trained using an *L*_2_-norm-based loss, while LFT (*L*_1_) adopts an *L*_1_-norm-oriented objective.

To simulate outlier contamination in the training data, we introduce additive noise to a subset of observed entries. Specifically, each entry in the training set is independently selected with probability ρ (the outlier rate) to be perturbed. For the chosen entries, noise *v* is sampled from a Gaussian mixture model whose probability density function is:

(15) $$p_{v}\left(v\right)=\sum_{i=1}^{2}\frac{c_{i}}{\sqrt{2\pi }\sigma_{i}}\exp\left(-\frac{v^{2}}{2\sigma_{i}^{2}}\right)$$

where $c_{1}+c_{2}=1$ with $0<c_{i}<1$ and $\sigma_{i}^{2}$ is variance. To simulate impulse noise, we use $\sigma_{2}^{2}>>\sigma_{1}^{2}$ and $c_{2}<c_{1}$, which means that large noise drawn from variance $\sigma_{2}^{2}$ and weight $c_{2}$ is embedded in Gaussian background noise with small variance $\sigma_{1}^{2}$. In our simulations, we set $\sigma_{2}^{2}=100\sigma_{1}^{2}$ and $c_{2}$ = 0.1. Consequently, the corrupted observation is defined as:

(16) $${\tilde{y}}_{ijk}=y_{ijk}+v,$$

where $y_{ijk}$ is the original entry and ${\tilde{y}}_{ijk}$ the noisy version. This construction effectively embeds sparse outliers within Gaussian background noise.

**Figure 5:**
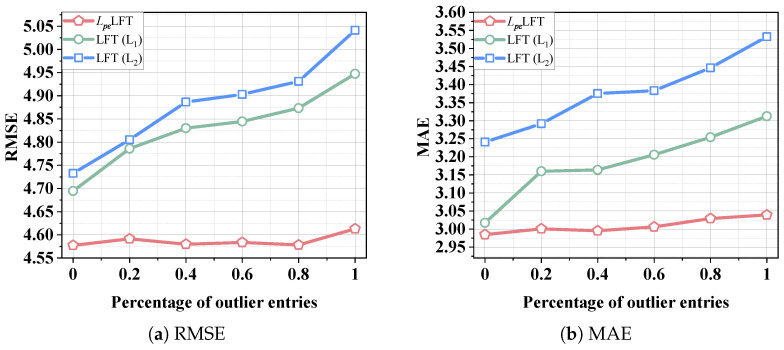
The outlier data sensitivity tests result of *L_p,ϵ_*LFT and LFT on D1.

Since *L_p,ϵ_*-norm exhibits lower sensitivity to outlier data than *L*_2_-norm and *L*_1_-norm, we find that *L_p,ϵ_*LFT becomes much more robust than LFT (*L*_1_) and LFT (*L*_2_) as the percentage of outlier data increases. The experimental results on D1, are displayed in Figure 5. The RMSE of *L_p,ϵ_*LFT and LFT (*L*_2_) are 4.5772 and 4.7327 without outliers and rise to 4.6131 and 5.0412 when all data are contaminated. The RMSE improvement for LFT (*L*_2_) is 0.3085, roughly 8.59 times larger than the 0.0359 improvement observed for *L_p,ϵ_*LFT. Furthermore, the results clearly indicate that, compared with the *L*_2_-norm, the *L_p,ϵ_*-norm achieves better performance under impulse noise.

### 5.4. Ablation Studies with the *L_p,ϵ_*LFT’s Hyper-Parameter Adaption Mechanism

This part of the experiments implements the ablation studies regarding the *L_p,ϵ_*LFT’s hyper-parameter adaption mechanism relying on the fuzzy control. With them, we aim to show that *L_p,ϵ_*LFT’s hyper-parameter adaption mechanism guarantees its high efficiency without accuracy loss. It is worth noting that removing the hyper-parameter adaption mechanism from *L_p,ϵ_*LFT yields the *L_p,ϵ_*LFT_manual_ model, where the subscript ’manual’ indicates that the hyper-parameters are determined through a manual grid search.

Table 7 presents the comparative performance results. These outcomes clearly demonstrate the beneficial effects of the self-adaptive hyper-parameters. Based on these observations, several important conclusions can be inferred.

1. ***L**_p,ϵ_***LFT incorporates a hyper-parameter adaptation mechanism that maintains its representation learning ability for a nonstandard tensor, ensuring no adverse impact on performance.** As recorded in Table 7, the RMSE/MAE gap between *L_p,ϵ_*LFT and *L_p,ϵ_*LFT_manual_, (i.e., (${\mathrm{RMSE}}_{\mathrm{high}}-{\mathrm{RMSE}}_{\mathrm{low}}$)/${\mathrm{RMSE}}_{\mathrm{high}}$ and (${\mathrm{MAE}}_{\mathrm{high}}-{\mathrm{MAE}}_{\mathrm{low}}$)/${\mathrm{MAE}}_{\mathrm{high}}$), is almost negligible. Namely, on dataset D1, the RMSE values of *L_p,ϵ_*LFT and *L_p,ϵ_*LFT_manual_ are 4.5563 and 4.5627, respectively, corresponding to a gap of 0.14%. Similarly, the MAE values are 2.9907 and 2.9773, respectively, with a gap of 0.44%. It is worth noting that comparable results can be observed across other test cases. Therefore, we can confidently conclude that the hyper-parameter adaptation mechanism in *L_p,ϵ_*LFT does not have adverse effects on its representation learning ability.
2. **Self-adaptive hyper-parameters significantly enhance** ***L**_p,ϵ_***LFT’s computational efficiency.** Notably, the cumulative computational cost of executing the learning model should include the time spent on hyper-parameter tuning to achieve optimal performance. For *L_p,ϵ_*LFT_manual_, the hyper-parameters *p*, ϵ, η, and λ require a four-fold grid search to determine their optimal values; therefore, the total computational cost comprises the cumulative training time under various hyper-parameter settings. In contrast, for a model employing a hyper-parameters adaptation mechanism, the total computational cost corresponds to a single execution since no explicit hyper-parameter tuning is needed. As shown in Table 7, it is evident that the total computational cost of *L_p,ϵ_*LFT is significantly lower than that of *L_p,ϵ_*LFT_manual_. For example, as displayed in Table 7, on D1, *L_p,ϵ_*LFT requires 67 s, which accounts for approximately 0.15% of the 42,500 s required by *L_p,ϵ_*LFT_manual_ in terms of RMSE. Similar results are observed across other datasets.
3. **To summarize, the hyper-parameter adaptation mechanism in** ***L**_p,ϵ_***LFT is essential, as it significantly improves efficiency and scalability without compromising representation accuracy.**

### 5.5. Comparison with State-of-the-Art Models

We evaluate *L_p,ϵ_*LFT in terms of both estimation accuracy and computational efficiency with multiple state-of-the-art models. Table 8 provides a concise description of all compared models. For M1, the hyper-parameters are adaptively tuned by the fuzzy controller, and the corresponding fuzzy rules are presented in Table 4.

For models M2-M7, details of the hyper-parameter settings adopted in our study are presented in Table 9. Models M1-M3 and M6-M7 are run on a computer equipped with a 3.40 GHz Intel i7 CPU and 32 GB RAM, whereas model M4 is executed on a GPU platform equipped with an NVIDIA GeForce RTX 4070 Ti SUPER GPU due to their higher computational demands.

**Figure 6:**
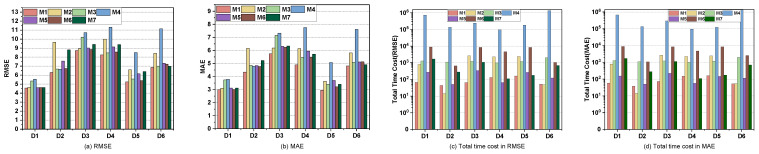
Performance comparison of M1–M7 on D1–D6.

The experimental results on datasets D1-6 for models M1-7 are shown in Table 10, with Figure 6 providing a visual illustration. Their computational efficiency is summarized in Table 11. Several key conclusions can be drawn from the previously presented experimental findings.

1. **The proposed** ***L**_p,ϵ_***LFT model (referred to as M1) demonstrates superior performance compared to other methods in predicting missing entries within the nonstandard tensor.** As illustrated in Figure 6 and summarized in Table 10, M1 consistently achieves the lowest RMSE and MAE on D1–6. Specifically, on D1, M1 attains an RMSE of 4.5563, which is about 1.95% lower than 4.6472 by M2 (RMSE_M2_-RMSE_M1_/RMSE_M2_), 14.87% lower than 5.3524 by M3, 18.08% lower than 5.5620 by M4, 1.23% lower by 4.6131 by M5, 1.67% lower than 4.6340 by M6, and 1.51% lower than 4.6263 by M7. In view of MAE, on D1, the output by M1–7 are 2.9907, 3.1008, 3.7351, 3.7896, 3.1170, 3.0029, and 3.1244, respectively. Consequently, M1’s MAE is also significantly lower compared to that of the other models. Consistent observations are found on D2–D6, with detailed results presented in Table 10 and Figure 6.
2. **The M1 exhibits markedly higher computational performance compared to its peers.** When evaluating the time requirements for a learning model to reach optimal performance, it is essential to consider the time cost of hyper-parameter tuning through grid search, particularly in the absence of self-adaptation for parameters. The cost is influenced by the number of hyper-parameters and empirical heuristics. Table 9 presents a summary of the optimal hyper-parameters values for M2–M7 obtained through grid search. As shown in Table 9, the fuzzy controller enables M1 to eliminate the need for grid search, thereby significantly enhancing its computational efficiency. As illustrated in Table 11 and Figure 6, on D1, the total time costs for RMSE and MAE for M1 are 67 and 58 s. These values are the lowest among all models, demonstrating that M1 achieves the lowest RMSE and MAE in a single training run. By comparison, for M6, the empirical range for Cauchy loss γ is typically [10, 400], and the regularization coefficient λ is usually [10^−1^, 10^−5^]. Obtaining optimally tuned results requires an average of 25 training iterations using a 5 × 5 hyper-parameter grid search. Thus, M6 requires approximately 8725 s to attain the minimum RMSE and MAE, indicating a noticeably greater computational cost compared with M1. Similarly, for D1, M2–M5 and M7 also exhibit higher time consumption in both RMSE and MAE evaluations than M1. This pattern consistently appears across D2–D6, as detailed in Table 11 and illustrated in Figure 6.
3. **The performance gains of M1 are substantial.** To further analyze these results, we conduct a Wilcoxon signed-ranks test on the data. This test, a nonparametric method for pairwise comparison, involves three key indicators: R+, R−, and the *p*-value. A greater R+ coefficient indicates better model performance, while the *p*-value assesses the statistical significance of the obtained outcomes. The findings of the Wilcoxon signed-rank tests at a 0.1 significance threshold are outlined in Table 12. Note that for Table 10 and Table 11, two distinct cases are considered, with RMSE and MAE as accuracy metrics for each dataset, leading to a total of 12 cases corresponding to 7 models.

As shown in Table 12, M1, i.e., an *L_p,ϵ_*LFT model, achieves significant gains in both accuracy and efficiency over its peers, as indicated by its consistently high R+ and *p*-values. Moreover, it shows superior results in all testing cases, as evidenced by 9 zeros for the *R*-value out of a total of 12 comparisons. Exceptions occur, as shown in Table 12, where M1’s computational cost is slightly higher than that of M2 on D2, M5 on D4, and M7 on D4. However, M1 significantly outperforms them in prediction accuracy. For instance, on D2, the RMSEs of M1 and M2 are 6.2902 and 9.6501, respectively, indicating that M1’s RMSE is about 34.82% lower than that of M2. On D4, the RMSEs of M1 and M5 are 8.2548 and 9.1325, respectively, showing that M1’s RMSE is approximately 9.61% lower than that of M5. These results suggest that the computational cost of M1 is acceptable when compared with state-of-the-art models.

### 5.6. Block-Wise Missing

This mode simulates temporal contiguous missing (TM) caused by faulty devices. The experiments are conducted by selecting a subset of temporal fibers and removing consecutive time intervals within each selected fiber.

Table 13 presents the reconstruction errors under the TM mode, where 50% of temporal fibers are randomly selected. The missing rate within each fiber ranges from 0.3 to 0.7. Under the TM mode, *L_p,ϵ_*LFT achieves superior estimation accuracy, demonstrating the effectiveness of the *L_p,ϵ_*-norm metric. For example, on the Guangzhou speed dataset, *L_p,ϵ_*LFT achieves the lowest RMSE of 4.7804 at a missing rate of 0.7, which is approximately 8.02% lower than that of M2 (5.1971), 19.36% lower than M3 (5.9281), 14.92% lower than M4 (5.6191), 5.97% lower than M5 (5.0841), 1.61% lower than M6 (4.8587), and 2.84% lower than M7 (4.9203). In addition, *L_p,ϵ_*LFT demonstrates greater stability than other methods, particularly at higher missing rates. These results indicate that *L_p,ϵ_*LFT is more robust to temporally contiguous missing data.

### 5.7. Case Study

**Figure 7:**
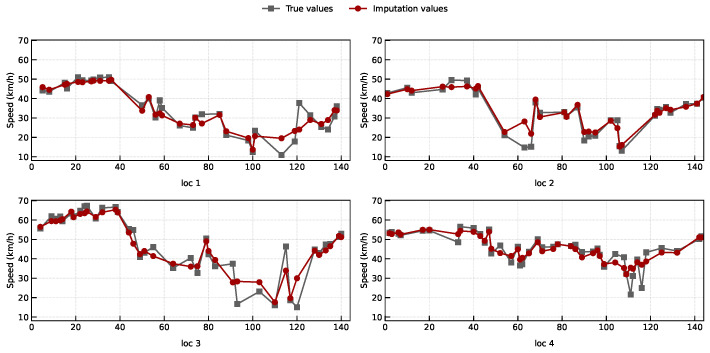
A case study to show the traffic speed time series, data imputation results on 4 sampling locations over a range of 1 day in the Guangzhou (D1) dataset.

In order to show the accuracy of the proposed *L_p,ϵ_*LFT model, we give a case study to visualize the imputation results for 4 randomly selected sampling locations over a range of one day in the D1 dataset in Figure 7. The grey line denotes the original traffic data, while the red line denotes the imputation data by our *L_p,ϵ_*LFT model. It is clear that the two lines are very close, and the distance between the imputed points and the true values is small.

### 5.8. Summary

Based on empirical analysis, the proposed *L_p,ϵ_*LFT model exhibits a range of advantages.

1. It constructs a generalized objective function based on the *L_p,ϵ_*-norm, which robustly represents outliers arising in real-world applications. Additionally, it estimates the LF matrices based on the observed data of the nonstandard tensor, thereby maintaining a computational efficiency that is competitive.
2. It incorporates an effective adaptation of hyper-parameters using a fuzzy controller, eliminating the need for costly manual tuning, and, thus, ensuring high scalability in real-world scenarios.

## 6. Conclusions

In this study, we introduce the Latent Factorization of Tensors model incorporating the *L_p,ϵ_*-norm (*L_p,ϵ_*LFT), aiming to recover incomplete traffic data in urban road networks. The model employs the *L_p,ϵ_*-norm within its objective function to enhance robustness and further integrates a fuzzy controller to fine-tune parameters efficiently. Experimental results on six real-world traffic datasets demonstrate that the proposed *L_p,ϵ_*LFT model achieves superior performance over seven state-of-the-art models in traffic data imputation, with an average RMSE reduction of 2.51% relative to the best-performing baseline. For future work, we plan to investigate adaptive regularization strategies and the design of more sophisticated objective functions to further strengthen model performance. These aspects will be systematically addressed in subsequent research.

## Figures and Tables

**Table 1:** Adopted symbols and their descriptions.

Symbol	Description
I,J,K	Number of sensors, time intervals, and days, respectively.
R	Real number domain.
Y	Original incomplete data tensor from ITS.
$\hat{Y}$	Low-rank approximation to Y.
X	A third-order latent factor tensor.
$X_{k}$	The *k*-th frontal slice of X.
U,S,T	Latent factor matrices.
$y_{ijk},{\hat{y}}_{ijk},x_{ijk}$	A single element in Y, $\hat{Y}$, X.
$u_{i},s_{j},t_{k}$	The *i*th, *j*th and *k*th row vectors of U, S, T.
$u_{ir},s_{jr},t_{tr}$	Single entries in U, S, T.
*R*	The dimension of latent factor space.
∘	Outer product of two vectors.
⊙	Hadamard product of two vectors.
${∥\cdot ∥}_{F}$	Frobenius norm of a tensor.
|·|	Cardinality of an enclosed set.
η	Learning rate.
λ	Regularization coefficient.
Λ(i),Λ(j),Λ(k)	The subsets of known entry set Λ related to entities ∀i∈I,∀j∈J,∀k∈K
K,Ψ,Ω	Training, validation and testing sets from Λ.

**Table 2:** Designed fuzzy rules.

$I^{\left(t\right)}$	*p*	ϵ	η	λ
$I_{1}$	$p_{1}$	$\epsilon_{1}$	$\eta_{1}$	$\lambda_{1}$
$I_{2}$	$p_{2}$	$\epsilon_{2}$	$\eta_{2}$	$\lambda_{2}$
$I_{3}$	$p_{3}$	$\epsilon_{3}$	$\eta_{3}$	$\lambda_{3}$
$I_{4}$	$p_{4}$	$\epsilon_{4}$	$\eta_{4}$	$\lambda_{4}$
$I_{5}$	$p_{5}$	$\epsilon_{5}$	$\eta_{5}$	$\lambda_{5}$

**Table 3:** Dataset details.

No.	Dataset	Sensor Count	Time Slots	Day Count	Known Entries
D1	Guangzhou	214	144	61	92,779
D2	Seattle	20,833	24	31	70,528
D3	New York	135	288	73	109,651
D4	Metr-la	207	288	119	65,190
D5	Pems-bay	325	288	181	84,685
D6	Berlin	12,416	24	31	138,999

**Table 4:** One-dimensional fuzzy rule.

$I^{\left(t\right)}$	*p*	ϵ	η	λ
1	0.42	0.9	0.0012	0.008
0.1	0.41	0.7	0.0011	0.009
0.01	0.40	0.5	0.0010	0.010
0.001	0.39	0.3	0.0009	0.011
0.0001	0.38	0.1	0.0008	0.012

**Table 5:** RMSE of *L_p,ϵ_*LFT_manual_ with optimal *p* and ϵ versus *L*_1_-norm and *L*_2_-norm.

No.	Optimal *p* and ϵ	EMSE	RMSE ($L_{1}$)	RMSE ($L_{2}$)
D1	p=0.6,ϵ=1	**4.5627 **	4.6946	4.7327
D2	p=0.4,ϵ=0.8	**6.2802**	6.6612	6.6074
D3	p=0.4,ϵ=0.8	**8.7366**	9.6978	9.1774
D4	p=0.4,ϵ=0.9	**8.2733**	8.8564	9.2885
D5	p=0.4,ϵ=1	**5.2792**	5.5320	6.1070
D6	p=0.2,ϵ=0.1	**6.8552**	7.1036	6.9729
Statistic	win/loss	6/0	0/6	0/6
	F-rank	1.00	2.50	2.50
	*p*-value	-	0.0156	0.0156

**Table 6:** MAE of *L_p,ϵ_*LFT_manual_ with optimal *p* and ϵ versus *L*_1_-norm and *L*_2_-norm.

No.	Optimal *p* and ϵ	MAE	MAE ($L_{1}$)	MAE ($L_{2}$)
D1	p=0.6,ϵ=1	**2.9773**	3.0172	3.2409
D2	p=0.4,ϵ=0.8	**4.3067**	4.6066	4.7001
D3	p=0.4,ϵ=0.8	5.7432	**5.6411**	6.4518
D4	p=0.4,ϵ=0.9	**4.8907**	5.0968	6.2254
D5	p=0.4,ϵ=1	**2.9584**	3.0126	3.8430
D6	p=0.2,ϵ=0.1	**4.8499**	4.9308	5.0583
Statistic	win/loss	5/1	1/5	0/6
	F-rank	1.17	1.83	3.00
	*p*-value	-	0.1094	0.0156

Bold values indicate the minimum MAE among the compared methods.

**Table 7:** Performance of *L_p,ϵ_*LFT with manually tuned and self-adaptive hyper-parameter.

Datasets	Metrics	*L_p,ϵ_*LFT	*L_p,ϵ_*LFT_manual_
D1	RMSE	**4.5563_±1.24 × 10^−2^_**	4.5627_±1.48 × 10^−2^_
	Time-RMSE	**67**_±14_	42,500_±8_
	MAE	2.9907_±1.46 × 10^−2^_	**2.9773**_±9.50 × 10^−3^_
	Time-MAE	**58**_±10_	47,500_±10_
D2	RMSE	**6.2902_±3.81 × 10^−2^_**	6.3532_±7.90 × 10^−2^_
	Time-RMSE	**43**_±2.19_	26,875_±7.42_
	MAE	**4.3322**_±1.66 × 10^−2^_	4.3483_±1.12 × 10^−2^_
	Time-MAE	**38**_±1.49_	26,875_±5.13_
D3	RMSE	**8.7279 _±2.61 × 10^−2^_**	8.7366_±1.51 × 10^−2^_
	Time-RMSE	**66**_±3.58_	37,500_±5.49_
	MAE	**5.7348_±7.55 × 10^−3^_**	5.7432_±4.99 × 10^−3^_
	Time-MAE	**72**_±8.56_	53,125_±5.80_
D4	RMSE	**8.2548**_±3.83 × 10^−2^_	8.2733_±3.22 × 10^−2^_
	Time-RMSE	**132**_±12.56_	63,750_±15_
	MAE	4.8916_±1.95 × 10^−2^_	**4.8907**_±1.52 × 10^−2^_
	Time-MAE	**149**_±10.43_	72,500_±4_
D5	RMSE	**5.2565**_±2.75 × 10^−2^_	5.2792_±1.65 × 10^−2^_
	Time-RMSE	**162**_±10_	95,000_±2.21_
	MAE	**2.9560**_±2.26 × 10^−2^_	2.9584_±2.99 × 10^−2^_
	Time-MAE	**162**_±10_	96,875_±0.14_
D6	RMSE	6.8597_±3.05 × 10^−3^_	**6.8552_±2.27 × 10^−2^_**
	Time-RMSE	**52**_±4.65_	135,000_±6.29_
	MAE	**4.8122_±1.05 × 10^−2^_**	4.8499_±6.44 × 10^−3^_
	Time-MAE	**53**_±4.39_	128,125_±7.20_

The bold values indicate higher prediction accuracy and lower total time cost. Note that the total computational time includes the duration of hyper-parameter tuning.

**Table 8:** Details of the compared models.

No.	Model	Description	Hyper-Parameter
M1	*L_p,ϵ_*LFT	The proposed model developed in this study.	Self-adaptation
M2	SGCP	A learning framework employing a sparse and graph constraint within a canonical polyadic (CP) tensor decomposition structure [36].	$\lambda_{1}$, sparse regularization $\lambda_{2}$, graph regularization
M3	SPTC	A smooth Poisson tensor completion algorithm that generalizes CP decomposition and incorporates the numerical priors of the data [37].	ρ, smoothing coefficient η, learning rate
M4	ENTED	An effective nonparametric tensor decomposition approach that utilizes a Gaussian process along with Pólya-Gamma augmentation to form conjugate models [38].	*p*, number of inducing points ζ, number of successes η, learning rate *M*, batch size
M5	BNLFT	A biased non-negative latent factorization of tensors for time-aware prediction [39].	λ, $\lambda_{b}$ regularization parameter
M6	CTF	A method based on CP decomposition, where the Cauchy loss function is used to quantify the difference between observed and predicted values [40].	γ, Cauchy loss λ, regularization coefficient
M7	TCA	A reverse CP decomposition method that updates all factor matrices using either alternating least squares or gradient descent techniques [41].	λ, regularization coefficient η, learning rate

**Table 9:** Hyper-parameter settings of M1–7 on D1–6.

Dataset	Hyper-Parameter Setting
D1	M1: Self-adaptive M2: $\lambda_{1}$ = 1, $\lambda_{2}$ = 1 M3: η = 1×10^−4^, ρ = 10 M4: *p* = 30, ζ = 10, η = 0.01, *M* = 64 M5: λ = 0.1, $\lambda_{b}$ = 0.1 M6: γ = 10, λ = 10^−3^ M7: η = 10^−5^, λ = 10^−2^
D2	M1: Self-adaptation M2: $\lambda_{1}$ = 1, $\lambda_{2}$ = 10 M3: η = 1×10^−4^, ρ = 50 M4: *p* = 10, ζ = 30, η = 0.01, *M* = 64 M5: λ = 1, $\lambda_{b}$ = 10^−6^ M6: γ = 10, λ = 10^−2^ M7: η = 10^−5^, λ = 10^−5^
D3	M1: Self-adaptation M2: $\lambda_{1}$ = 1, $\lambda_{2}$ = 1000 M3: η = 1×10^−4^, ρ = 70 M4: *p* = 10, ζ = 50, η = 0.01, *M* = 128 M5: λ = 0.1, $\lambda_{b}$ = 0.1 M6: γ = 200, λ = 10^−5^ M7: η = 10^−5^, λ = 10^−1^
D4	M1: Self-adaptive M2: $\lambda_{1}$ = 0.1, $\lambda_{2}$ = 1000 M3: η = 1×10^−3^, ρ = 10 M4: *p* = 10, ζ = 30, η = 0.01, *M* = 64 M5: λ = 1, $\lambda_{b}$ = 10^−4^ M6: γ = 300, λ = 10^−5^ M7: η = 10^−4^, λ = 10^−1^
D5	M1: Self-adaptive M2: $\lambda_{1}$ = 1, $\lambda_{2}$ = 1000 M3: η = 1×10^−3^, ρ = 10 M4: *p* = 30, ζ = 50, η = 0.01, *M* = 128 M5:λ = 0.1, $\lambda_{b}$ = 10^−5^ M6: γ = 200, λ = 10^−5^ M7: η = 10^−4^, λ = 10^−1^
D6	M1: Self-adaptive M2: $\lambda_{1}$ = 1, $\lambda_{2}$ = 10 M3: η = 1×10^−4^, ρ = 30 M4: *p* = 30, ζ = 10, η = 0.01, *M* = 64 M5: λ = 1, $\lambda_{b}$ = 10^−3^ M6: γ = 300, λ = 10^−5^ M7: η = 10^−5^, λ = 10^−2^

**Table 10:** Prediction accuracy (RMSE and MAE) of all tested models on D1–6.

Case		M1	M2	M3	M4	M5	M6	M7	Gains vs. Best
D1	**RMSE**	**4.5563_±1.24 × 10^−2^_**	4.6472_±2.06 × 10^−2^_	5.3524_±2.10 × 10^−2^_	5.5620_±0_	4.6131_±1.73 × 10^−2^_	4.6340_±6.21 × 10^−3^_	4.6263_±1.04 × 10^−2^_	1.23%
	**MAE**	**2.9907_±1.46 × 10^−2^_**	3.1008_±1.38 × 10^−2^_	3.7351_±2.41 × 10^−2^_	3.7896_±0_	3.1170_±1.47 × 10^−2^_	3.0029_±4.05 × 10^−3^_	3.1244_±6.07 × 10^−3^_	0.41%
D2	**RMSE**	**6.2902_±3.81 × 10^−2^_**	9.6501_±2.00 × 10^−1^_	6.6815_±1.91 × 10^−1^_	6.6626_±0_	7.5726_±3.81 × 10^−2^_	6.7513_±1.54 × 10^−2^_	8.8178_±2.96 × 10^−3^_	5.59%
	**MAE**	**4.3322_±1.66 × 10^−3^_**	6.1528_±1.60 × 10^−1^_	4.8513_±1.53 × 10^−1^_	4.7785_±0_	4.8338_±2.54 × 10^−2^_	4.7716_±1.88 × 10^−2^_	5.2233_±4.55 × 10^−4^_	9.21%
D3	**RMSE**	**8.7279_±2.61 × 10^−2^_**	8.9452_±2.27 × 10^−2^_	10.2218_±1.35 × 10^−1^_	10.7175_±0_	9.0236_±3.44 × 10^−2^_	8.8570_±1.14 × 10^−2^_	9.4179_±3.88 × 10^−2^_	1.46%
	**MAE**	**5.7348_±7.55 × 10^−3^_**	6.1897_±6.79 × 10^−3^_	7.1542_±2.20 × 10^−1^_	7.3256_±0_	6.3108_±3.42 × 10^−2^_	6.2360_±1.51 × 10^−2^_	6.3332_±1.56 × 10^−2^_	7.89%
D4	**RMSE**	**8.2548_±4.17 × 10^−2^_**	10.0005_±5.30 × 10^−2^_	8.4697_±2.15 × 10^−2^_	11.3228_±0_	9.1325_±1.60 × 10^−1^_	8.5762_±4.38 × 10^−2^_	9.3954_±4.26 × 10^−2^_	2.54%
	**MAE**	**4.8916_±4.34 × 10^−2^_**	6.1495_±4.74 × 10^−2^_	5.4630_±1.05 × 10^−2^_	7.7476_±0_	5.9487_±7.74 × 10^−2^_	5.4852_±4.94 × 10^−2^_	5.7196_±4.18 × 10^−2^_	10.46%
D5	**RMSE**	**5.2565_±2.75 × 10^−2^_**	6.6003_±3.45 × 10^−2^_	5.5713_±3.00 × 10^−2^_	8.5231_±0_	6.1571_±1.65 × 10^−2^_	5.3868_±2.81 × 10^−2^_	6.4094_±4.36 × 10^−2^_	2.42%
	**MAE**	**2.9560_±2.26 × 10^−2^_**	3.6441_±2.27 × 10^−2^_	3.4066_±2.04 × 10^−2^_	5.0772_±0_	3.7010_±8.07 × 10^−3^_	3.2087_±2.66 × 10^−2^_	3.4008_±6.07 × 10^−2^_	7.87%
D6	**RMSE**	**6.8597_±3.05 × 10^−2^_**	8.4374_±1.98 × 10^−2^_	7.0087_±2.49 × 10^−2^_	11.1509_±0_	7.3273_±4.76 × 10^−2^_	7.2214_±3.25 × 10^−2^_	6.9866_±7.33 × 10^−3^_	1.82%
	**MAE**	**4.8122_±1.05 × 10^−2^_**	5.8063_±2.66 × 10^−2^_	5.0891_±2.26 × 10^−2^_	7.6089_±0_	5.1150_±3.99 × 10^−2^_	5.1257_±1.34 × 10^−2^_	4.9100_±8.73 × 10^−3^_	1.99%

The underlined number represents the minimum value of the comparison model for each metric (RMSE or MAE). Gains vs. Best indicates the percentage improvement of our model over the best-performing baseline across each metric.

**Table 11:** Time costs of all tested models on D1–6.

Case		M1	M2	M3	M4	M5	M6	M7
D1	Time-RMSE	**67_±14_**	786_±0.78_	1275_±4.55_	721,875_±45_	273 _±1.38_	8725_±16.74_	1700_±1.94_
	Time-MAE	**58_±10_**	798_±0.83_	1275_±4.55_	664,375_±43_	151_±1.03_	8725_±16.74_	1700_±1.86_
D2	Time-RMSE	43_±2.19_	**14**_±8.11 × 10^−2^_	1100_±3.46_	135,000_±10_	50_±0.79_	650_±6.79_	275_±0.53_
	Time-MAE	38_±1.49_	**14**_±8.38 × 10^−2^_	1100_±3.46_	135,000_±10_	50_±0.79_	1050_±15.12_	275_±0.47_
D3	Time-RMSE	**66_±3.58_**	2632_±1.49_	1200_±2.87_	239,375_±28_	341_±2.96_	8375_±2.07_	1075_±2.41_
	Time-MAE	**72_±8.56_**	2677_±0.94_	1200_±2.87_	290,000_±41_	224_±1.91_	8375_±2.07_	1125_±2.59_
D4	Time-RMSE	132_±10.81_	2240_±5.34_	975_±3.44_	95,000_±34_	**66**_±2.28_	4625_±9.00_	111_±0.20_
	Time-MAE	149_±9_	2245_±5.34_	975_±3.44_	95,000_±34_	**57**_±2.60_	4750_±9.40_	109_±0.21_
D5	Time-RMSE	**162_±10_**	2400_±0.83_	1175_±1.48_	181,250_±24_	263_±0.30_	8400_±0.58_	178_±0.32_
	Time-MAE	**162_±10_**	2408_±0.83_	1175_±1.48_	122,500_±31_	145_±1.24_	8400_±0.58_	172_±0.20_
D6	Time-RMSE	52_±4.65_	**49**_±5.65_	2000_±2.87_	1,323,750_±24_	121_±0.10_	1050_±3.17_	675_±1.68_
	Time-MAE	**53**_±4.39_	54_±8.03_	2000_±2.87_	1,323,750_±24_	114_±0.27_	2575_±33.61_	700_±1.71_

**Table 12:** Results of the Wilcoxon signed-rank test.

Comparison	Accuracy			Efficiency		
	**R+**	**R−**	***p*** **-Value**	**R+**	**R−**	***p*** **-Value**
M1 vs. M2	78	0	0.0002	69	9	0.0080
M1 vs. M3	78	0	0.0002	78	0	0.0002
M1 vs. M4	78	0	0.0002	78	0	0.0002
M1 vs. M5	78	0	0.0002	63	15	0.0319
M1 vs. M6	78	0	0.0002	78	0	0.0002
M1 vs. M7	78	0	0.0002	71	7	0.0046

**Table 13:** Prediction accuracy (RMSE and MAE) of all tested models with TM on Guangzhou speed data.

Missing Rate		M1	M2	M3	M4	M5	M6	M7
0.3	**RMSE**	**4.6052**	4.7912	5.5672	5.5936	4.7043	4.6694	4.7248
	**MAE**	**2.9845**	3.2276	3.9178	3.8178	3.1997	3.0387	3.1974
0.5	**RMSE**	**4.6830**	4.9424	5.7674	6.8441	4.8746	4.7645	4.8171
	**MAE**	**3.0316**	3.3052	4.1162	4.9200	3.3273	3.1243	3.2609
0.7	**RMSE**	**4.7804**	5.1971	5.9281	5.6191	5.0841	4.8587	4.9203
	**MAE**	**3.1496**	3.5681	4.2584	3.8087	3.5056	3.2218	3.3343
Statistic	win/loss	6/0	0/6	0/6	0/6	0/6	0/6	0/6
	F-rank	1.000	4.833	6.500	6.500	4.000	2.000	3.167
	*p*-value	–	0.0156	0.0156	0.0156	0.0156	0.0156	0.0156

## Data Availability

The raw data supporting the conclusions of this article will be made available by the authors on request.

## Appendix A. Comparison Between *L_p,ϵ_*-Norm and *L_p_*-Norm

We now demonstrate that the *L_p,ϵ_*-norm serves as a lower bound for the *L_p_*-norm when 0<p≤2, while the analysis is conducted using a two-element case for simplicity, the argument can be generalized to vectors of arbitrary length. Construct g($e_{1}$, $e_{2}$) as:

(A1) ge1,e2=ep,εp−epp=e12+ε2pp22−εp−e1p+e22+ε2pp22−εp−e2p.

Noting that g($e_{1}$$,e_{2}$) is an even function, it suffices to verify that g($e_{1}$$,e_{2}$)≤0 under the condition $e_{1}>0$ and $e_{2}>0$. By applying the inequality of $\sqrt{a^{2}+b^{2}}\leq a+b$ valid for all non-negative *a* and *b*, we derive the following:

(A2) $$\begin{array}{c} g\left(e_{1},e_{2}\right)\leq h\left(e_{1},e_{2}\right)={\left(e_{1}+\varepsilon \right)}^{p}-\varepsilon^{p}-e_{1}^{p}+{\left(e_{2}+\varepsilon \right)}^{p}-\varepsilon^{p}-e_{2}^{p}. \end{array}$$

For *p* = 1, it is easy to get:

(A3) $$h\left(e_{1},e_{2}\right)=\left(e_{1}+\varepsilon \right)-\varepsilon -e_{1}+\left(e_{2}+\varepsilon \right)-\varepsilon -e_{2}=0,$$

which means ${∥e∥}_{1,\varepsilon }^{1}\leq {∥e∥}_{1}^{1}$ where the equal sign holds if and only if e=0. When 0<p<2, the partial derivatives of the h$\left(e_{1},e_{2}\right)$ with respect to each variable are given by:

(A4) $$p\left({\left(e_{1}+\varepsilon \right)}^{p-1}-e_{1}^{p-1}+{\left(e_{2}+\varepsilon \right)}^{p-1}-e_{2}^{p-1}\right),$$

which is negative when $e_{i}>0$, it follows that h$\left(e_{1},e_{2}\right)\leq h\left(0,0\right)=0$. Together with the inequality g$(e_{1},e_{2})\leq$ h($e_{1},e_{2}$), we deduce that g$(e_{1},e_{2})\leq$0, indicating ${∥e∥}_{p,\varepsilon }^{p}\leq {∥e∥}_{p}^{p}$ where the equality holds exclusively when e=0. This establishes that the *L_p,ϵ_*-norm serves as the lower convex envelope of the *L_p_*-norm for 0<p≤2.
